# Fluoxetine during Development Reverses the Effects of Prenatal Stress on Depressive-Like Behavior and Hippocampal Neurogenesis in Adolescence

**DOI:** 10.1371/journal.pone.0024003

**Published:** 2011-09-01

**Authors:** Ine Rayen, Daniël L. van den Hove, Jos Prickaerts, Harry W. Steinbusch, Jodi L. Pawluski

**Affiliations:** Department of Neuroscience, School of Mental Health and Neuroscience, Maastricht University, Maastricht, The Netherlands; University of South Florida, United States of America

## Abstract

Depression during pregnancy and the postpartum period is a growing health problem, which affects up to 20% of women. Currently, selective serotonin reuptake inhibitor (SSRIs) medications are commonly used for treatment of maternal depression. Unfortunately, there is very little research on the long-term effect of maternal depression and perinatal SSRI exposure on offspring development. Therefore, the aim of this study was to determine the role of exposure to fluoxetine during development on affective-like behaviors and hippocampal neurogenesis in adolescent offspring in a rodent model of maternal depression. To do this, gestationally stressed and non-stressed Sprague-Dawley rat dams were treated with either fluoxetine (5 mg/kg/day) or vehicle beginning on postnatal day 1 (P1). Adolescent male and female offspring were divided into 4 groups: 1) prenatal stress+fluoxetine exposure, 2) prenatal stress+vehicle, 3) fluoxetine exposure alone, and 4) vehicle alone. Adolescent offspring were assessed for anxiety-like behavior using the Open Field Test and depressive-like behavior using the Forced Swim Test. Brains were analyzed for endogenous markers of hippocampal neurogenesis via immunohistochemistry. Results demonstrate that maternal fluoxetine exposure reverses the reduction in immobility evident in prenatally stressed adolescent offspring. In addition, maternal fluoxetine exposure reverses the decrease in hippocampal cell proliferation and neurogenesis in maternally stressed adolescent offspring. This research provides important evidence on the long-term effect of fluoxetine exposure during development in a model of maternal adversity.

## Introduction

Depression during pregnancy and the postpartum period is a growing health concern that affects up to 20% of women [1], [2], [3], [4]. Maternal stress, depression and anxiety, can have long-term effects on the physical and mental development of children [5], [6], [7]. For example, antenatal maternal depression can lead to neurobehavioral disturbances, such as impaired cognitive and social developmental outcomes [6], [7], [8], [9], [10], [11]. In rodent models, stress during gestation, which results in depressive-like behavior in the dam [12], [13], models the clinical findings [11], [14]. Several animal studies have indicated that adult offspring of prenatally stressed mothers show increases in affective-related behavior [14], [15], [16], [17] and decreased levels of hippocampal neurogenesis [18], [19], [20], [21]. Given the development effect of exposure to maternal depression, it is crucial to treat this disorder in order to improve maternal and child outcomes.

Selective serotonin reuptake inhibitor (SSRIs) medications are commonly used for the treatment of maternal depression [22]. Current estimates suggest that there is an increasing incidence of SSRI use in mothers that ranges between 5–10% [23], [24], [25]. However, the effects of these medications on the developing child have yet to be fully determined [5], [26]. Recent clinical studies report that neonates exposed to SSRI medications during gestation, regardless of maternal mood state, have an increased risk for low birth weight, younger gestational age, neurobehavioral disturbances, and reduced heart rate variability [5], [27], [28]. Recent evidence also demonstrates that prenatal exposure to SSRI-medications may alter neurodevelopment as evidenced via alterations in S100B levels [29]. In addition, perinatal exposure to SSRI medications may have long term effects on mood in children [30], [31]. For example, children perinatally exposed to maternal depression and SSRIs exhibit increased internalizing behaviors at 3 years [31].

Preclinical data is beginning to show that exposure to SSRIs during development significantly impacts offspring affective-like behaviors and neural plasticity [5], [26], [32]. For example, SSRI treatment, via intraperitoneal (i.p.) injection to offspring, during the early postnatal period can result in increased depressive- and anxiety-like behavior during adulthood [33], [34], [35]. Developmental exposure to SSRIs may also influence neuroplasticity in the hippocampus, through effects on brain derived neurotrophic factor (BDNF) mRNA levels [34].

Although these studies point to a role for SSRIs in development, it should be noted that in preclinical studies offspring are treated with or exposed to SSRIs alone, and not in combination with maternal depression. To date, very little research has looked at the effect of maternal stress and SSRIs on offspring outcomes [36], [37] and only one study has combined both a model of maternal stress and fluoxetine exposure [36]. This study demonstrated that postnatal oral administration of the SSRI, fluoxetine, to pups reverses the stress induced reduction in CA3 spine and synapse density in juveniles and young adults [36]. In this study, SSRI treatment alone, in the absence of maternal stress, had no affect on spine density measures in the CA3 region of the hippocampus [36]. Thus the actions of early exposure to SSRI medications may be very different in the presence of maternal adversity. Therefore to better translate these findings to the clinic, the effects of maternal use of SSRI medications need to be investigated in animal models of maternal adversity.

The aim of the present study was to investigate the developmental effect of fluoxetine, a popular SSRI antidepressant used during pregnancy, in a model of maternal adversity, on anxiety and depression-related behavior and hippocampal neurogenesis in adolescent male and female offspring. Although research has investigated the developmental impact of perinatal SSRI exposure on offspring outcomes, little research has been done on the neurodevelopmental effects of postnatal fluoxetine treatment in an animal model of maternal depression. In addition, much less research has looked at the long-term effects of developmental SSRI exposure during adolescence, a time of vulnerability to stress [38], [39], [40], [41], [42]. Our data shows that the exposure to fluoxetine during development can reverse the effect of prenatal stress on aspects of adolescent development. Knowledge of the effects of maternal depression and antidepressant treatment during the perinatal period is needed to ameliorate treatment and intervention options, and thus improve neurodevelopmental outcomes.

## Methods

### Animals

Twenty-two adult female Sprague-Dawley rats (250–300 g; Charles River Laboratories, France) were used in the present study. Rats were kept under standard laboratory conditions in a 12h∶12h light/dark schedule (lights on at 07:00 h) with *ad libitum* access to rat chow (Sniff) and tap water. All experiments were approved by the Animal Ethics Board of Maastricht University in accordance with Dutch governmental regulations (approval IDs: DEC 2008-157 and DEC 2008-158). All efforts were made to minimize the pain and stress levels experienced by the animals.

On gestation day (GD) 15, dams were randomly assigned to stress (n = 12) or control groups (n = 10). Dams in the stress group were individually restrained three times a day for 45 min in transparent plastic cylinders under bright light (between 8–10am, 12–2pm, 4–6pm) on GD15–20 and twice on GD21 as previously described [43], [44]. This time period during pregnancy is when stress can result in postpartum depressive-like behavior in the dam [12], [13] and a period of stress that affects offspring outcomes [17], [45].

**Figure 1:**
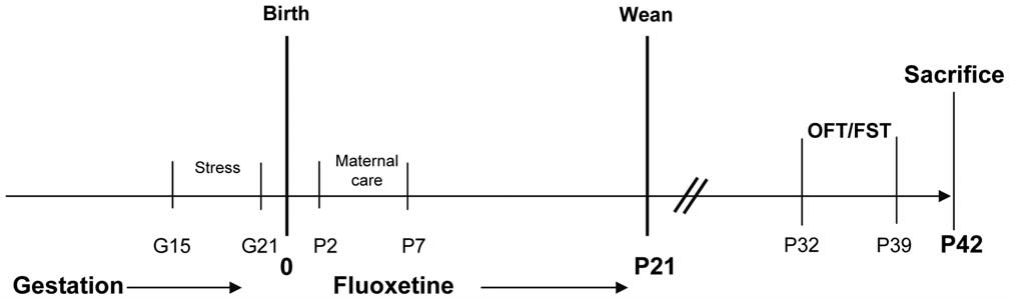
Timeline of experiment. Stress was administered between GD15-21. Fluoxetine treatment to the mother began 1 day after birth and continued until weaning (P21). Between P32 and P39, offspring were subjected to behavioral tasks. At P42, offspring were sacrificed.

One day after birth (birth day = P0), litters were culled to 5 males and 5 females and dams (with offspring) were randomly assigned to one of two treatment groups: fluoxetine (5 mg/kg/day) or vehicle, for a total of four groups of dams: 1) Prenatal Stress + Vehicle (PSV; n = 5), 2) Prenatal Stress + Fluoxetine (PSF; n = 7), 3) Control + Fluoxetine (CF; n = 5), and 4) Control + Vehicle (CV; n = 5). A maximum of 2 male and 2 female offspring per litter were used in the present experiment (n = 9–11/sex/group). Offspring litter was weighed on P21 and individual weights were taken once between P29–31 and once between P39–42. For assessment of hippocampal cell proliferation and neurogenesis, 5 animals per group were used (1 male and 1 female from each litter). For a time line of the experiment see Figure 1.

### Fluoxetine treatment

Fluoxetine treatment was administered via osmotic minipumps (Alzet Osmotic pumps, 2ML4) to the dam on P1. Fluoxetine and its active metabolite, norfluoxetine, can pass to offspring through lactation [46], therefore we used this mode of delivery to prevent the stress of administration via injection, or oral gavage to the offspring. In addition, rodent brain development during the early postpartum period is analogous to human brain development during the third trimester [47].

Implants were filled with either fluoxetine (Fagron, Belgium), dissolved in vehicle (50% propylenediol in saline; 5 mg/kg/day), or with vehicle as previously described [48]. Minipumps were implanted subcutaneously in the dorsal region while the dams were under mild isofluorene anesthesia on post-partum day 1 (P1).

### Maternal care

Maternal care was assessed twice a day for 5 minutes from P2 to P7 based on previous literature [49]. Scoring took place in the morning (between 8:30 a.m. and 10:30 a.m.) and the afternoon (between 13:30 p.m. and 15:30 p.m.) with at least 3 h between the sessions. During each testing period the duration of the following maternal behaviors was assessed: licking (licking/grooming; licking/grooming/nursing), nursing (arched-back nursing, “blanket” nursing, and passive nursing) and nest building. Data were aggregated across days and were calculated as total percent time spent in each behavior.

### The Open Field Test (OFT)

The OFT was used to study anxiety-like behavior and locomotor activity in adolescent offspring [50]. The open field test consisted of a 100 cm×100 cm area divided into central and peripheral areas with 40 cm high walls. For the test, a rat was placed in the centre of the field and behavior was recorded for five minutes. All animals were tested once between 9:30 a.m. and 2 p.m. (age P32–34). A video-tracking system (Anymaze, Stoelting) was used to score the distance travelled, number of entries into the central and peripheral areas, and total time spent in the central and peripheral areas. The apparatus was cleaned with 70% ethanol and dried between rats.

### The Forced Swim Test (FST)

The forced swim test (FST) was used to assess depressive-like behavior in the adolescent offspring as previously described [51], [52], [53]. The apparatus consisted of a vertical cylindrical glass tank (height 50 cm × diameter 20 cm) filled to a depth of 20 cm with tap water at 27 ± 1°C. For the test, an animal was placed in a cylindrical glass tank for 10 min. Offspring were tested on the FST, between 9 a.m. and 1 p.m. (age P37–39). Using the Best Collection System (Educational Consulting Inc.), behaviors scored in the FST were (1) immobility – floating with the absence of any movement and (2) struggling – quick movements of the forelimbs such that the front paws break the surface of the water.

### Immunohistochemistry (IHC)

A minimum of 2 days after the last behavioral test, offspring were deeply anesthetised with an overdose of pentobarbital, and decapitated. Half of the brain was used for IHC, the hippocampus of the other half was used for further analysis not included in the present study. Brains were post-fixed in 4% paraformaldehyde for 48 hours, cryoprotected in 30% sucrose/phosphate-buffered saline solution for up to one week, frozen on dry ice and kept at −80°C. Brain tissue was sliced in 40 µm sections on a cryostat (Leica). Tissue was stored in antifreeze solution and maintained at −15°C. The number of proliferating cells and immature neurons were assessed in the dentate gyrus of the hippocampus using endogenous markers, i.e. Ki67 for cell proliferation and doublecortin (DCX) for immature neurons. Every 6^th^ section throughout half the hippocampus was stained as previously described [54], [55]. Sections were blocked with H_2_O_2_ and incubated overnight in either rabbit anti-Ki67 (1∶500; Vector Laboratories) or goat anti-DCX (1∶200; Santa Cruz). Sections were then incubated overnight in biotinylated donkey anti-rabbit (1∶500; Jackson ImmunoResearch, Suffolk, UK) or for 2 hours in biotinylated rabbit anti-goat (1∶500; Jackson ImmunoResearch) secondary antibody. Brain sections were further processed by using the avidine-biotine complex (ABC Elite kit; 1∶1000; Vector laboratories). DAB (3,3-diaminobenzidine; Sigma) was used as a substrate to obtain a color reaction. Sections were mounted on gelatin-coated slides, dried overnight, counterstained with Cresyl Violet acetate, dehydrated and coverslipped with Permount (Fisher Scientific).

**Figure 2:**
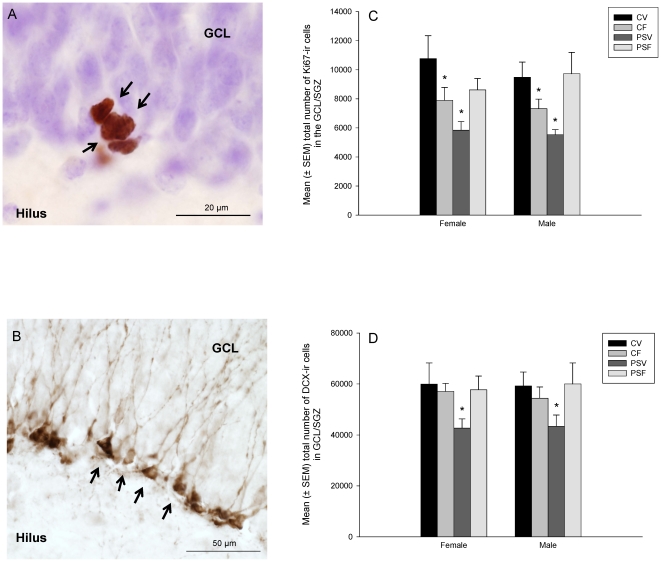
Photomicrographs of representative A) Ki67-ir cells and B) DCX-ir cells in the GCL/SGZ and mean (± SEM) number of C) Ki67-ir cells and D) DCX-ir cells in the GCL/SGZ. C) PSV adolescent offspring had significantly fewer Ki-67-ir cells in the GCL/SVZ compared to all other groups (.0001≤p≤.01). CF adolescent offspring had significantly more Ki67-ir cells in the GCL/SVZ compared to PSV offspring (p≤.0001), but significantly fewer Ki67-ir cells in the GCL/SVZ compared to CV and PSF offspring (.002≤p≤.04), regardless of sex. D) PSV adolescent offspring had significantly fewer number of DCX-ir cells in the GCL/SGZ of the hippocampus compared to all other groups (.006≤p≤.03), regardless of sex. ‘*’denotes significantly different from all other groups. (n = 5/sex/group).

The number of Ki67 immunoreactive (-ir) cells and DCX-ir cells were counted under 40× objective with oil as previously described [54], [55]. Cells were considered Ki67-ir if they were intensely stained and exhibited medium round or oval cell bodies (Figure 2A). Cells were considered DCX-ir if they exhibited medium round or oval cell bodies and dendrites (Figure 2B). The areas of the granule cell layer/subgranular zone (GCL/SGZ) and hilus were measured using StereoInvestigator software (MicroBrightField, Williston, VT, USA) and estimates of GCL/SGZ and hilus volumes were made using Cavalieri's principle [56].

### Statistical analyses

Analysis of variance tests (ANOVA) were done for maternal behaviors with condition (prenatal stress/no stress) and treatment (fluoxetine/vehicle) as independent factors. ANOVAs were done on offspring weight gain, FST measures, OFT measures, Ki67-ir and DCX-ir cell numbers with condition (prenatal stress/no stress), treatment (fluoxetine/vehicle), and sex (male/female) as independent factors. Pearson product moment correlations were conducted between behaviors on the OFT (central entries, central time, and central distance) and FST (struggling and floating), and the total number of Ki67-ir and DCX-ir cells for all groups and separately by treatment and condition. Any differences in age, weight, time of testing or test order of the litter, were accounted for, where appropriate, via an analysis of covariance. In cases where clear sex differences were evident stratified analysis were done separately for each sex. *Posthoc* comparisons utilized the Fisher LSD test.

## Results

### Maternal care

Stressed dams spent a significantly greater percentage of time nest building compared to non-stressed dams, regardless of fluoxetine treatment (main effect of treatment; F(1, 18) = 10.66, p≤.004; Table 1). Independent of treatment condition, dams spent a significantly greater percentage of time nursing offspring than licking offspring (main effect of time; F(1, 18) = 358.37, p≤.00001; Table 1).

**Table 1:** Mean (± SEM) percentage of time in maternal behaviors.

	CV	CF	PSV	PSF
Licking (%)	11.00±2.68	6.92±1.68	10.56±3.03	7.82±1.89
Nursing (%)	71.5±5.84	79.19±6.70	73.14±4.06	72.98±5.06
Nest building (%)	0.36±0.12	0.28±0.13	2.78±0.89*	1.32±0.51*

Stressed dams spent a significantly greater percentage of time nest building compared to non-stressed dams, regardless of fluoxetine treatment (p≤.004). Regardless of treatment or condition, dams spent a significantly greater percentage of time nursing offspring than licking offspring (p≤.00001). ‘*’ denotes significantly different from CV and CF.

### Body weight change

**Figure 3:**
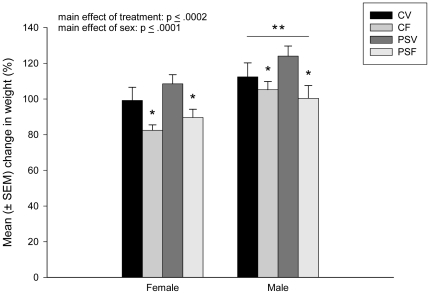
Mean (± SEM) percentage in weight change from P29 to P42. CF and PSF offspring gained significantly less weight than CV and PSV offspring, regardless of stress (p≤.0002). Overall, male adolescent offspring gained significantly more weight than female offspring (p≤.0001). ‘*’denotes CF and PSF significantly different from CV and PSV groups. ‘**’ denotes males significantly different from females. (n = 9–11/sex/group).

CF and PSF offspring gained significantly less weight than CV and PSV offspring, regardless of stress (main effect of treatment; F(1,68) = 15.33, p≤.0002; Figure 3). Overall, male offspring gained significantly more weight than female offspring (main effect of sex; F(1,68) = 16.71, p≤.0001; Figure 3), even when controlling for any age differences at the time of weighing.

### The OFT

**Figure 4:**
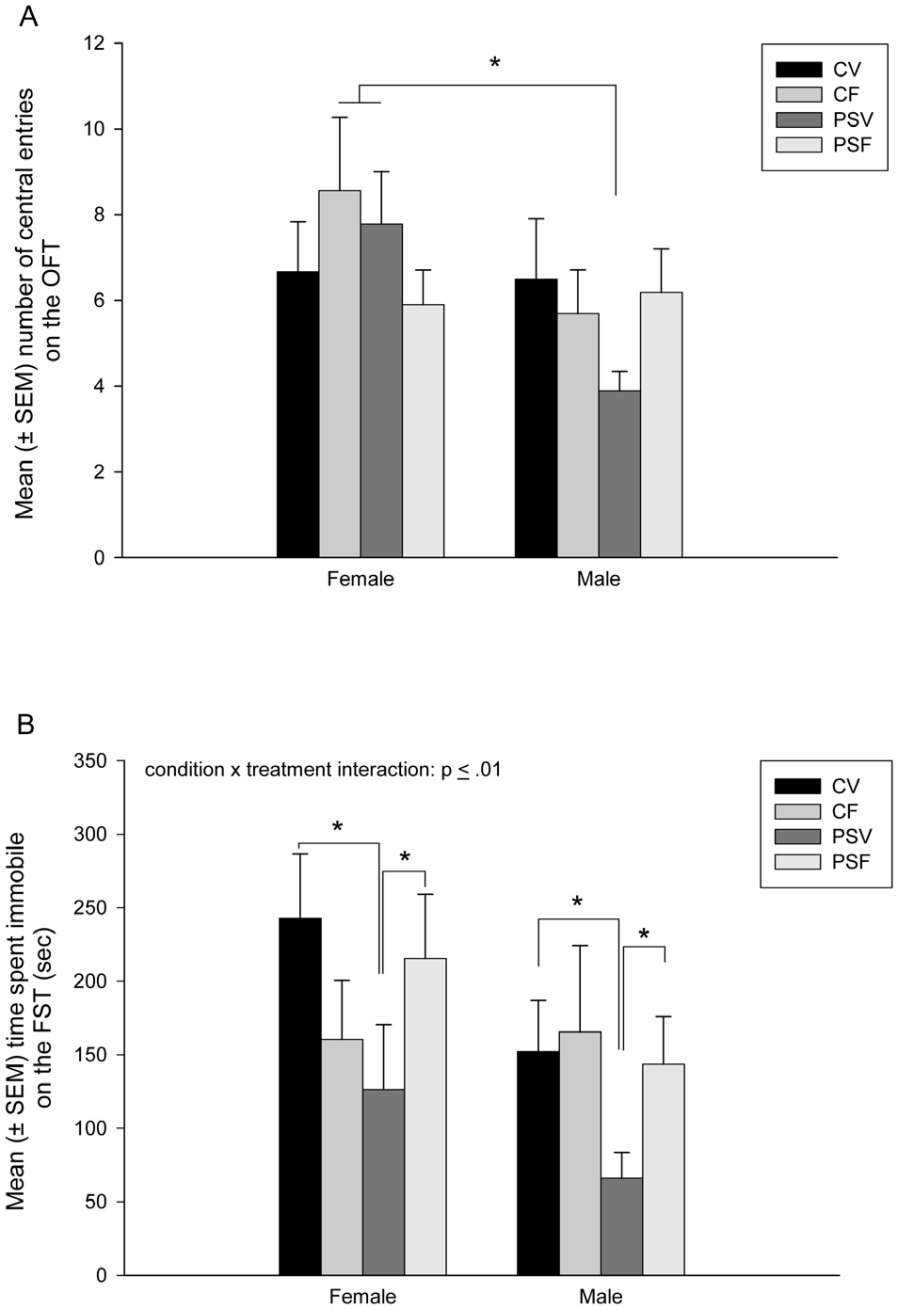
Mean (± SEM) A) number of central entries (OFT) and B) time spent immobile (FST). A) PSV male offspring made significantly fewer central entries compared to CF and PSV female offspring (.007≤p≤.02). There was also a significant main effect of sex with male offspring making significantly fewer central entries compared to female offspring (p≤.05). Further analysis by sex revealed that PSV males made fewer central entries than CV, CF and PSF adolescent males, however this did not reach significance (p≥0.09) and there were no significant effect of treatment or condition in female adolescent offspring (p≥0.14). B) PSV adolescent offspring spent significantly less time immobile compared to CV and PSF offspring (.02≤p≤.04). (n = 9–11/sex/group).

PSV male offspring made significantly fewer central entries compared to CF and PSV female offspring (.007≤p≤.02: condition×treatment×sex effect; F(1,69) = 4.43, p≤.04; Figure 4A). There was also a significant main effect of sex with male offspring making significantly fewer central entries compared to female offspring (F(1,69) = 4.14, p≤.05). Further analysis by sex revealed that PSV males made fewer central entries than CV, CF and PSF adolescent males, however this did not reach significance (p≥0.09) and there were no significant effects of treatment or condition in female adolescent offspring (p≥0.14). There were no other significant differences between groups in measures on the OFT (.07≤p≤.90; Table 2).

**Table 2:** Mean (± SEM) total distance and time in the centre of the OFT and percent time spent struggling during the FST.

	Female offspring				Male Offspring			
	CV	CF	PSV	PSF	CV	CF	PSV	PSF
**OFT:**								
Total distance (m)	24.46±1.84	27.48±1.71	26.93±2.15	21.85±2.63	24.41±2.71	22.32±1.99	22.80±1.76	24.41±1.77
Central distance (m)	2.36±.56	3.47±.65	2.86±.39	1.79±.36	1.87±.34	2.49±.39	1.79±.18	2.20±.53
Centre time (sec)	21.16±6.27	20.44±3.43	15.74±1.83	15.04±2.68	10.93±2.06	16.69±3.71	13.46±1.49	16.37±4.60
**FST:**								
Struggling (sec)	52.30±10.2	47.09±7.82	57.63±13.45	53.90±7.84	53.54±11.77	30.36±5.38	61.66±13.83	47.25±12.38

There were no significant differences between treatments, condition, or sex in total or central distance travelled on the OFT or amount of time spent in the centre of the OFT. There were also no significant differences between treatment, conditions, or sex in amount of time spent struggling in the FST (0.12≤p≤.90).

### The FST

PSV adolescent offspring spent significantly less time immobile compared to CV and PSF offspring (.02≤p≤.04: condition×treatment effect; F(1, 68) = 7.17, p≤.09, controlling for weight differences; Figure 4B). There were no significant differences between conditions, treatment or sex in amount of time spent struggling in the FST and no other significant main effects or interactions on measures of the FST (0.12≤p≤.90).

### Ki67-ir cells

There were no significant differences between groups in the volume of the GCL/SGZ and the hilus of the hippocampus (p>.07), therefore total number of Ki67-ir cell counts were used for statistical analysis. Results demonstrate that PSV adolescent offspring had significantly fewer Ki-67-ir cells in the GCL/SVZ compared to all other groups (.0001≤p≤.01). CF adolescent offspring had significantly more Ki67-ir cells in the GCL/SVZ compared to PSV offspring (p≤.0001), but significantly fewer Ki67-ir cells in the GCL/SVZ compared to CV and PSF offspring (.002≤p≤.04; condition × fluoxetine × region (GCL, hilus) effect; F(1, 32) = 14.58, p≤.0006; Figure 2C), regardless of sex. There was also a significant interaction effect between stress and fluoxetine (F(1,32) = 18.1, p≤.0002), a main effect of stress (F(1,32) = 4.18, p≤.05) and a significant effect of region with more Ki67-ir cells in the GCL/SVZ compared to the hilus (F(1,32) = 466.03, p≤.0001). There were no significant correlations between number of Ki67-ir in the GCL/SVZ and measures on the OFT or FST and no other significant main effects of interactions (0.07≤p≤.92).

### DCX-ir cells

PSV adolescent offspring had significantly fewer number of DCX-ir cells in the GCL/SGZ of the hippocampus compared to all other groups (.009≤p≤.03, condition × treatment effect; F(1,32) = 5.97, p≤.020; Figure 2D), regardless of sex. There was a significant negative correlation between number of DCX-ir cells in the GCL/SGZ and time spent in the centre of the OFT in PSV offspring (r = −.83, p≤.003) and a significant positive correlation between number of DCX-ir cells in the GCL/SVZ and amount of time spent struggling in the FST in CF offspring (r = .67, p≤.03). There were no other significant correlations between number of DCX-ir cells in the GCL/SVZ and measures on the OFT or FST and no other significant main effects of interactions (.07≤p≤.84).

## Discussion

The results of the present study demonstrate that early exposure to fluoxetine in combination with maternal stress has long-term effects on body weight, depressive-like behavior, and hippocampal neurogenesis in offspring. Our primary findings show that early postnatal exposure to maternal fluoxetine reversed the decrease in immobility in the FST, hippocampal cell proliferation and hippocampal neurogenesis in maternally stressed adolescent offspring. In addition, we found that fluoxetine exposure alone significantly reduced hippocampal cell proliferation in comparison to controls and maternally stressed offspring exposed to fluoxetine. We did not find any differences in pup-directed maternal care with fluoxetine treatment or maternal stress suggesting that our data point to a developmental impact of fluoxetine, in the presence of maternal adversity, on offspring outcomes.

### Developmental exposure to fluoxetine reduces weight gain in adolescent offspring

We found that postnatal fluoxetine exposure, regardless of exposure to prenatal maternal stress, significantly decreased post-weaning weight gain in adolescent male and female offspring. Previous research showed that a high dose of in utero fluoxetine, via drinking water to dams, resulted in a decrease of birth weight and also a reduction in weight gain during the pre-weaning period in rats [57]. Moreover, several studies have shown that postnatal treatment of fluoxetine, via injection to offspring, leads to a loss of body weight in adult mice and guinea pigs [34], [58], [59]. A reduction in weight gain during the pre-weaning period may be a result of the involvement of 5-HT in glucoregulation in the hypothalamus [60] such that high levels of 5-HT, as a result of the blockade of 5-HT reuptake by fluoxetine, may inhibit the ingestion of carbohydrates and, as a consequence, lead to weight loss [61].

### Prenatal stress and anxiety-like behavior in adolescent offspring

In the present study we found that adolescent male offspring had increased anxiety-like behaviors, as evident by fewer central entries in the open field test, compared to adolescent female offspring. We also report that prenatal stress increased anxiety-like behavior in the adolescent male, but only significantly different in comparison to prenatally stressed or fluoxetine-treated adolescent females. Although further work is needed on the effect of prenatal stress and maternal fluoxetine use on anxiety-like behavior of offspring during adolescence, these data are in partial agreement with work done in prenatally stressed adult offspring. For example, previous research has shown an increase in anxiety-like behavior in prenatally stressed adult male, but not female, offspring [62]. More recently work has also demonstrated an increase in anxiety-like behavior in prenatally stressed male offspring, using a similar maternal stress paradigm as in the present study [63].

We did not find an effect of developmental fluoxetine exposure on anxiety-like behavior in adolescent offspring. Previous work has also shown minimal effects of early fluoxetine exposure alone, via i.p. injections to pups from postnatal day 4–21, on anxiety-like behavior in adult male mice in the light-dark box or open field test [34]. However, more recent work has shown that administration of fluoxetine during gestation, to healthy non-stressed dams, results in increased anxiety-like behavior in adult male rats, as measured on the elevated plus maze [64]. Therefore, it seems likely that the effect of early exposure to SSRIs, on anxiety-like behavior later in life may depend on many factors which include the timing of the SSRI exposure, the timing of the test, and the test used to assess anxiety-like behavior.

We also did not find a marked relationship between anxiety-like behaviors and measures of hippocampal neurogenesis in the adolescent rats. Previous work has demonstrated that hippocampal neurogenesis is associated with anxiety-like behavior in adulthood; Revest et al (2009) demonstrated that transgenic mice with decreased levels of hippocampal neurogenesis had increased anxiety-like behavior [65]. However, more research is needed to determine the role of hippocampal neurogenesis in anxiety-like behavior during development. It should also be noted that the relationship between hippocampal neurogenesis and anxiety-like behavior in adolescent rats may significantly vary compared to that of transgenic adult mice as there are well known strain and species differences in hippocampal neurogenesis [66].

### Developmental exposure to fluoxetine reverses the effects of prenatal stress on immobility in adolescent offspring

In the present study we found that developmental fluoxetine exposure to prenatally stressed offspring reversed the decrease in immobility in the FST seen in adolescent offspring exposed to prenatal stress alone, while developmental fluoxetine exposure alone had no significant effect on immobility in adolescent offspring. Previous work on the effects of postnatal SSRI treatment on depressive-like behavior in offspring has shown that i.p. injection of SSRIs to offspring during development, in the absence of maternal stress, leads to increased immobility in the FST during adulthood [33], [67]. Others have shown that oral fluoxetine administration to the dam during pregnancy and lactation increases immobility in the forced swim test during adolescence (P30) and adulthood (P70) in female mouse offspring [68]. Discrepancies between our findings and others may be due to the timing and dose of fluoxetine administration, the species tested, and when, after weaning, animals were tested. For example, Lisboa et al (2007) found that fluoxetine exposure (7.5 mg/kg), via oral gavage, to mouse dams during pregnancy and lactation resulted in increased depressive-like behavior in female mouse offspring during adolescence, where as we administered fluoxetine (5 mg/kg) to rat dams during lactation only and tested rat offspring during adolescence. Therefore, the developmental impact of SSRIs may also depend on when during development the exposure occurred. It is also possible that other tests of depressive-like behavior, such as the sucrose preference test, may provide more insight in to the effects of stress and/or SSRIs on offspring behavior.

Although, the exact mechanisms by which fluoxetine counteracts the decrease in immobility in prenatally stressed adolescent offspring is not known, considerable evidence suggests that prenatal maternal stress programs the hypothalamic-pituitary-adrenal (HPA) axis as well as behavior, and that plasticity of the developing monoamine system in the brain underlies, in part, these changes [11], [69]. Furthermore, prenatal exposure to fluoxetine can alter HPA function [5], [36], [70], and thus may act to ‘regulate’ physiological systems impacted by early exposure to maternal adversity.

### Developmental exposure to fluoxetine increases hippocampal neurogenesis in prenatally stressed adolescent offspring

In the present study postnatal fluoxetine exposure to maternally stressed offspring reversed the decrease in hippocampal neurogenesis evident after prenatal stress. In addition, postnatal fluoxetine exposure alone decreased hippocampal cell proliferation but had no effect on hippocampal neurogenesis. During adulthood, chronic fluoxetine treatment can significantly upregulate hippocampal neurogenesis [71], [72]. However, our data suggests that developmental exposure to fluoxetine reverses the decrease in hippocampal cell proliferation and hippocampal neurogenesis in prenatally stressed offspring and returns the levels of hippocampal neurogenesis back to those of control animals. Interestingly, these data point to a long-term impact of developmental exposure to fluoxetine on hippocampal neurogenesis which are dependent on exposure to maternal adversity. Whether these changes in hippocampal cell proliferation and production of immature neurons impact hippocampal circuitry and behavioral correlates remains to be determined. Further work is also needed to investigate the persistence of the effects of maternal adversity and developmental exposure to fluoxetine on hippocampal neurogenesis in adult offspring.

The mechanism behind the effects of SSRI exposure on the developing hippocampus has yet to be determined, but developmental exposure to SSRIs have been reported to affect the developing serotonergic system [73], [74], and BDNF levels in the hippocampus [34]. For example, postnatal citalopram treatment, via subcutaneous injections to the pups (P8–21) can lead to a decrease in the serotonin transporter levels in the hippocampus of rat offspring [73]. In addition, postnatal treatment with SSRIs, via i.p. injections to the offspring, can lead to upregulation of BDNF mRNA in the hippocampus [34]. Thus exposure to SSRIs during development may act to alter hippocampal neurogenesis through its actions on many systems of the developing brain.

Our data also demonstrates that the action of fluoxetine on the hippocampal neurogenesis varies in the presence of maternal stress. Although most research to date has investigated the developmental impact of SSRIs in offspring of healthy mothers, one study has shown that early treatment with fluoxetine may act to ‘correct’ the effect of maternal stress on neuron morphology [36]. In this work Ishiwata et al (2005) demonstrated that postnatal SSRI administration, via oral administration of fluoxetine to pups, reverses the prenatal stress induced reduction in CA3 spine density at 3 and 9 weeks of age but SSRI treatment alone, in the absence of maternal stress, had no long-term effect on spine density measures in the CA3 region of the hippocampus of offspring [36]. As mentioned previously, it is likely that developmental exposure to fluoxetine in offspring exposed to maternal adversity, may act to regulate the HPA axis and thus ‘normalize’ the effect of glucocorticoids on hippocampal plasticity in prenatally stressed offspring. Further work is needed to investigate the mechanism of fluoxetine action on the developing brain in response to maternal adversity.

### Conclusions

A growing number of children are exposed to SSRI medications during perinatal development [5], yet our knowledge of the long-term impact of this drug exposure is limited. Findings from our work show that developmental exposure to maternal fluoxetine, in combination with exposure to prenatal maternal stress, reverses the effects of prenatal stress on depressive-like behavior and hippocampal neurogenesis in adolescent offspring. Thus, there may be a potential beneficial role of developmental exposure to fluoxetine in the presence of maternal adversity. However, before conclusions can be made much more work is needed not only in models of maternal adversity, but using other popular SSRIs, serotonin-norepinephrine reuptake inhibitors (SNRIs) and psychotropic medications being used to treat mood disorders during pregnancy and postpartum [32].

In conclusion, further preclinical work is needed to understand the long-term implications of developmental exposure to SSRIs and other antidepressant medications in the presence of maternal adversity before conclusions can be made about the use of antidepressant medications to treat maternal depression during the perinatal period.
